# A novel AhR ligand, 2AI, protects the retina from environmental stress

**DOI:** 10.1038/srep29025

**Published:** 2016-07-01

**Authors:** Mark A. Gutierrez, Sonnet S. Davis, Andrew Rosko, Steven M. Nguyen, Kylie P. Mitchell, Samiha Mateen, Joana Neves, Thelma Y. Garcia, Shaun Mooney, Gary H. Perdew, Troy D. Hubbard, Deepak A. Lamba, Arvind Ramanathan

**Affiliations:** 1 University of Denver, Colorado 2199 S University Blvd, Denver, CO 80208, USA; 2 Buck Institute for Research on Aging, 8001 Redwood Blvd, Novato, CA, 94901, USA; 3 University of Washington Box 358047 Seattle, WA 98195, USA; 4 The Pennsylvania State University, Center for Molecular Toxicology and Carcinogenesis, 309 Life Sciences Building, University Park, PA 16802, USA.

## Abstract

Various retinal degenerative diseases including dry and neovascular age-related macular degeneration (AMD), retinitis pigmentosa, and diabetic retinopathy are associated with the degeneration of the retinal pigmented epithelial (RPE) layer of the retina. This consequently results in the death of rod and cone photoreceptors that they support, structurally and functionally leading to legal or complete blindness. Therefore, developing therapeutic strategies to preserve cellular homeostasis in the RPE would be a favorable asset in the clinic. The aryl hydrocarbon receptor (AhR) is a conserved, environmental ligand-dependent, per ARNT-sim (PAS) domain containing bHLH transcription factor that mediates adaptive response to stress via its downstream transcriptional targets. Using *in silico, in vitro* and *in vivo* assays, we identified 2,2′-aminophenyl indole (2AI) as a potent synthetic ligand of AhR that protects RPE cells *in vitro* from lipid peroxidation cytotoxicity mediated by 4-hydroxynonenal (4HNE) as well as the retina *in vivo* from light-damage. Additionally, metabolic characterization of this molecule by LC-MS suggests that 2AI alters the lipid metabolism of RPE cells, enhancing the intracellular levels of palmitoleic acid. Finally, we show that, as a downstream effector of 2AI-mediated AhR activation, palmitoleic acid protects RPE cells from 4HNE-mediated stress, and light mediated retinal degeneration in mice.

Retinal pigmented epithelium (RPE) cells are important for maintaining intercellular homeostasis in the retina. These cells form a barrier through the formation of tight junctions between neighboring pigmented epithelial cells, controlling the amount of nutrients, ions, and fluids between the neuroretina and the choroid^1^,^2^. One of the more significantly noted features of the RPE is the capacity to phagocytose and metabolize outer segments that are shed by the light-sensitive rod and cone photoreceptors^3^,^4^. Dysregulation of this function has a potential to play a role in the degeneration of the retina^5^. Overall, it has been ascertained that the functional disruption and atrophy of the RPE is a key factor in the progression of degenerative conditions in the retina, leading to the death of other cell types in the retina, including the rod and cone photoreceptors, resulting in significant vision loss^6^,^7^. Therefore, developing strategies to maintain the function and cellular homeostasis of the RPE is a significant point of investigation with regards to preventing retinal degeneration in humans. In this context, the Aryl hydrocarbon receptor (AhR) has been implicated to play a role in maintaining retinal homeostasis^8^,^9^. This transcription factor is a ligand-dependent Per-ARNT-Sim (PAS)/bHLH transcription factor that has been originally identified as the receptor for 2,3,7,8-tetrachlorodibenzo-*p*-dioxin (referred to as dioxin) and then cloned in the 1990’s^10^. Since then, AhR is critical for cellular responses to environmental stimuli through the induction of detoxification factors such as the cytochrome P450 enzymes^11^. AhR is most notably known to be activated in response to environmental stressors such as xenobiotic stimuli. It has also been shown to be active in response to endogenous metabolic products such as those found in the tryptophan oxidation pathway such as 6-formylindolo[3,2-b]carbazole (FICZ) and kynurenic acid^12^.

It has also been shown that the knock-out of AhR in mice can lead to the degeneration of RPE cells in an age-dependent fashion^8^,^9^, suggesting a critical role for AhR in protecting RPE cells from chronic environmental stress. This indicates that molecules that activate AhR in degenerative conditions might promote retinal homeostasis. To study this we utilized *in vitro* assays to characterize the role of AhR signaling in RPE cell homeostasis. Canonical polyaromatic hydrocarbon ligands of AhR are not suitable drug candidates due to their numerous cytotoxic effects^10^. Based on previously known natural indole based ligands of AhR^13^, we identified a novel indole containing synthetic AhR-ligand 2,2′-aminophenyl indole (2AI) that potently induces the expression of the cytochrome P450, family 1a1, members (CYP1A1 and CYP1B1), and maintains RPE-cell viability in the presence of 4-hydroxynonenal (4HNE). Finally, we identified the omega-7 monounsaturated fatty acid commonly known as palmitoleic acid, as a downstream effector of 2AI, which we show to be protective against 4HNE treatment in human RPE cells and light-mediated toxicity in the murine retina.

## Results

### AhR is expressed and activated by light-induced stress in retina *in vivo*

**Figure 1:**
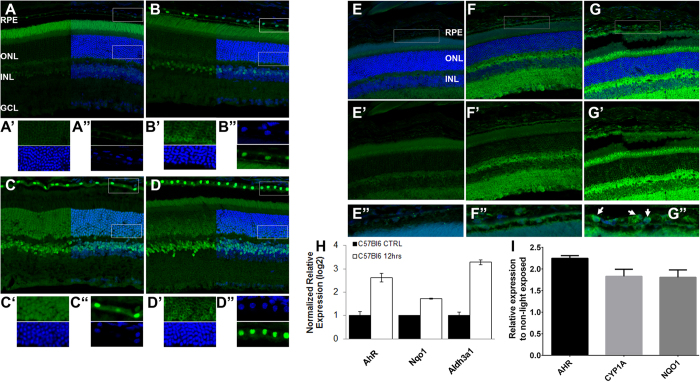
Light-Exposure Activates AhR Signaling in mammalian retina. (**A–D**) AhR staining of light exposed retinas. Retinal sections from C57Bl6 (control (**A**) and 6 hours after light (**B**)) and Balb/C mice (control (**C**) and 6 hours after light (**D**)) stained with DAPI (nuclear stain, blue) and an anti-AhR antibody (green) showing nuclear translocation of AhR. A’, A”, B’, B”, C’, C”, D’ and D” show zoomed-in views of the boxed regions in (**A–D**) demonstrating co-localization of AhR and DAPI in RPE and photoreceptor cells. (**E–G**) CYP1A staining (in green) of control (E-E”) and light exposed C57Bl6 retinas at 6 hours (F-F”) and at 24 hours (G-G”). E”, F” and G” are zoomed-in views of boxed regions in (**E–G**) and arrows in G” mark RPE cells expressing CYP1A. DAPI (blue) stains nuclei (**H**) Relative mRNA-levels of AhR targets in murine neural retina 12 hrs after light exposure. (**I**) Relative expression of AhR and its targets in hESC-derived RPE cells 12 hours after *in vitro* blue light (400 nm) exposure for 30–60 mins. (Scale bar = 20 μm).

Previous studies have implicated light-induced damage in the progression of retinal degeneration focusing on the consequences of light-induced damage on cell function and homeostasis in the retina, noting changes in neural morphology, photoreceptor responsiveness, along with the induction of apoptosis^14^,^15^,^16^. It is well known that a consequence of photo-oxidative stress is an increase in the production of reactive lipid aldehydes, primarily 4HNE, leading to lipid peroxidation of retinal tissues and cytotoxic protein modifications in retinal cells^17^. Knock-out of AhR in mice leads to progressive retinal degeneration^9^ and in human retinas, there is a progressive decline in AhR signaling without a measurable change in AhR abundance^9^. To test the direct role of AhR in retinal stress response, 6–8 week old C57Bl6 mice were stressed using a previously established light damage paradigm by exposing them to 10klux of white light for 1 hour. Mice were analyzed 6–24 hours following light exposure. In undamaged adult retinas, we observed AhR expression in mature photoreceptors and was localized in the outer segment region consistent with previous reports of AhR upregulation as photoreceptors mature^8^. High-intensity light exposure in C57Bl6 results in a clear translocation of AhR into the nucleus of photoreceptors and RPE cells as well as some cells in the inner nuclear layer of the retina (Fig. 1A–A”). We next analyzed Balb/C mice which are under constant light stress^18^. In these mice, even under basal conditions, we observe robust nuclear AhR expression in both the RPE and the photoreceptor cells as well as the inner retina (Fig. 1C–C”). Interestingly, following exposure to light conditions which lead to photoreceptor apoptosis in the Balb/C mice (Fig. S1A), there is a change in the localization of AhR in the photoreceptor cells (Fig. 1D–D’) with a concomitant increase in the photoreceptor segments while the RPE layer still expressed high-levels of AhR which was localized in the nuclei while the inner retina expression is unchanged (Fig. 1D–D”). This was associated with a concomitant decrease in AhR gene expression at 24 hours in light-exposed Balb/C mice by qPCR (Fig. S1B). This suggests that promoting the activity of the AhR might play a role in chemo-protection against light-induced stress. To test if the AhR nuclear translocation was associated with AhR effector activity, we analyzed CYP1A protein expression, a well characterized AhR-response gene, by IHC at 6 and 24 hours following light damage (Fig. 1E–G). We observed CYP1A expression in the neural retina within 6 hours while RPE expression was observed at 24 hours following light stress. We further confirmed AhR activity in the neural retina by qPCR in light exposed C57Bl6 mice at 12 hours. We observed up regulation of AhR, and two of its known down-stream targets *Nqo1* and *Aldh3a1*, both of which participate in detoxification of cells (Fig. 1H). Similarly, AhR and its effectors (CYP1A and NQO1) expression was increased in hESC-derived RPE cells *in vitro* following 30–60 mins of blue light (400 nm) exposure (Fig. 1I). Though the blue light-stress maybe different from full-wavelength light used in mouse studies, it is more closely associated with AMD progression in some studies^19^ and has previously been used to study stress *in vitro*^20^,^21^. This suggests that AhR activity is a physiological response to light stress.

It is known that photo-oxidation of tryptophan can generate a number of indole containing compounds such as FICZ that can bind to AhR. These endogenous ligands are short lived, but represent a class of compounds that could inspire a new ligand, that can be used to activate AhR therapeutically. Here we investigated whether novel indole-containing synthetic ligands that activate AhR might help to protect the retina and RPE.

### *In silico* screening leads to identification of 2AI, a novel indole based synthetic ligands that activates the AhR pathway

**Figure 2:**
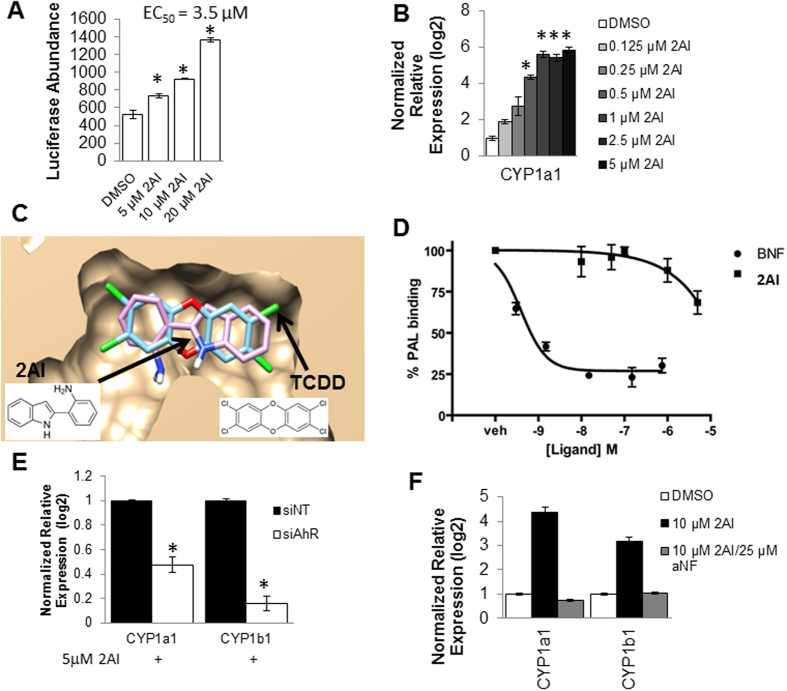
2AI activates AhR Signaling in ARPE19 cells (**A**) 2AI activates luciferase activity (luminescence units on Y axis) of an AhR binding, XRE-luciferase reporter assay in a concentration dependent manner (concentrations displayed on X axis). (**B**) mRNA levels (from ARPE19 cells) of CYP1a1, an AhR target measured using qPCR treated with varying concentration of 2AI. (**C**) Comparison of binding of 2AI as compared with the known AhR ligand TCDD from *in silico* docking. (**D**) Photo-affinity ligand competition assay using humanized AhR liver cytosol (% photoaffinity label bound the protein) vs. concentrations of 2AI and positive control (BNF). (**E**) siRNA mediated knockdown of AhR or the untargeted siNT control, followed by relative mRNA expression of AhR targets CYP1a1 and CYP1a2 with or without treatment with 5 μM 2AI (**F**) mRNA levels of CYP1a1 following treatment with 2AI or co-treatment with AhR antagonist aNF. (n = 3, *p < 0.05).

AhR is activated in response to environmental stress, either from xenobiotic or endogenous chemical ligands^22^. The environmental chemicals that modulate AhR signaling arise from (i) synthetic chemicals, or xenobiotics derived from (ii) dietary plants and (iii) microbes. The structural diversity of its ligands suggests that this transcription factor can respond to an array of environmental signals. Of particular interest are indole containing organic compounds that are a produced by microbiome, plants and mammalian metabolic pathways. Phytometabolites like indole-3-carbinol have also been known to activate AhR^23^,^24^. We hypothesized that synthetic compounds containing the indole scaffold could act as novel ligands of AhR which mimic physiological indole based ligands. Such compounds based on natural scaffolds might have advantages of increased bioavailability, safety, and compatibility over traditional poly aromatic organic compound based ligands. We carried out an *in silico* screen of approximately 70 indole containing compounds (filtered from 2000 commercially available compounds from Sigma Aldrich) to identify potent ligands of AhR (workflow is outlined in Fig. S2 and supplementary methods). The screen resulted in the identification of a novel ligand, 2AI, as judged by a luciferase reporter assay (Fig. 2A; EC_50_ = 3.5 μM), and increased the expression of known AhR targets CYP1A1 in ARPE19 cells, a human RPE cell line, in a concentration dependent fashion (Fig. 2B). *In silico* docking image of 2AI in comparison with the AhR ligand TCDD is shown in Fig. 2C. We validated the direct binding of 2AI to AhR using a photo-affinity ligand based competition assay (Fig. 2D) as described previously^25^,^26^. 2AI was able to out-compete the photoaffinity label bound to the human AhR protein at micro molar concentrations (with beta naphthoflavone (BNF) used as a positive control). In our experience small molecular weight and hydrophilic compounds like 2AI can often show rapid off rate kinetics, leading to the underestimation of their binding to the AhR protein *in vitro*. This is because canonical ligands of AhR such as TCDD and BNF used in experiments tend to be polyaromatic and highly hydrophobic and so tend to bind irreversibly while hydrophilic ligands bind reversibly. Finally, we characterized AhR dependence of the ligand using siRNA mediated knockdown of AhR in ARPE19 cells followed by treatment with 5 μM 2AI (Figs 2E and S3A). siRNA mediated knockdown of AhR prevented the 2AI mediated increase in the AhR targets *Cyp1a1* and *Cyp1b1*, but this was not observed in the non-targeting siNT control (Fig. 2E). This shows that the 2AI signals via AhR to activate canonical AhR transcriptional targets. Further, the co-treatment of ARPE19 cells with established AhR antagonists alpha-naphthoflavone (*a*NF) (Fig. 2F) and CH223191 (Fig. S3C) prevented the 2AI dependent increase in expression of *Cyp1a1* expression. The above data validates the *in silico* screening platform and the discovered novel ligand of AhR, 2AI.

### TCDD and 2AI activate the AhR pathway and protect against 4HNE induced cytotoxicity in human RPE cells

**Figure 3:**
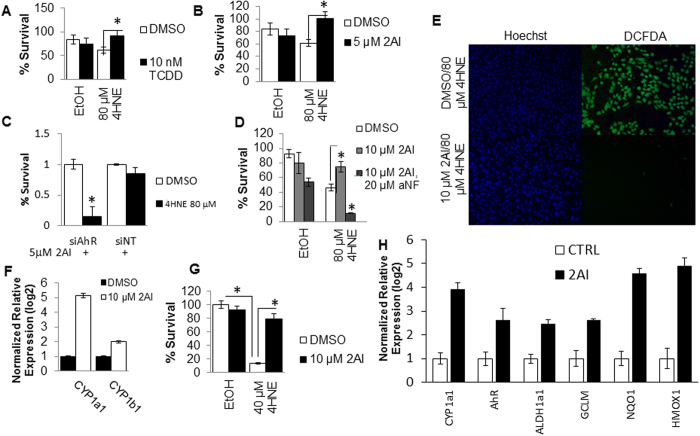
2AI activates AhR Signaling in RPE cells and Protects from 4HNE induced toxicity. (**A**) Viability of 4HNE treated ARPE19 cells in the presence or absence of TCDD. (**B**) Viability of 4HNE treated ARPE19 cells in the presence or absence of 2AI. (**C**) % survival of RPE 4HNE treated cells, with or without 2AI, and either control siNT or siAhR knockdown. (**D**) % survival of cells treated with 4HNE with or without 2AI in the presence or absence of the AhR antagonist aNF. (**E**) 4HNE treated cells in the presence or absence of 2AI, stained with DAPI and DCFDA. (**F**) Relative mRNA levels of CYP1a1 and CYP1b1 after treatment of human ESC derived RPE cells with 2AI for 48 hrs. (**G**) % survival of 4HNE treated human ESC derived RPE cells in presence of absence of 2AI. (**H**) Up regulation in the expression of AhR and its effectors (CYP1A1, ALDH1a1) as well as NRF2 effectors (GCLM, NQO1 and HMOX1) in human ESC-derived RPE cells by QT-PCR following stimulation with AhR activator, 2AI (10 μM for 24 hours). (n = 3, *p < 0.05).

4HNE is one of the major end-products of lipid peroxidation and is a known inducer of oxidative stress in various tissues^27^. Here, we characterized the ability of 2AI to provide cellular protection against 4HNE induced cytotoxicity using the well characterized ligand tetrachlorodibenzodioxin (TCDD)^13^ as control. TCDD is a potent ligand of AhR that is formed by incomplete combustion of fuels and industrial wastes. It is associated with a number of toxic effect such as cholracne^28^, therefore there is a need to identify drugable physiological ligands of AhR. All assays were done in RPE cells grown in 96 well plates, and viability was assessed by measuring intracellular ATP levels. Both TCDD (in Fig. 3A, 10 nM) and 2AI (in Fig. 3B, 5 μM based on Fig. 2B) protect ARPE19 cells, from 4HNE mediated toxicity (IC_50_ = 80 μM and Fig. S3D). We also confirmed the protective effects of 2AI using two different viability dyes- (Calcein-AM) which measures total cellular esterase activity (Fig. S3E), and ethidium bromide which is incorporated selectively by dead cells (Fig. S3F). We next tested the effects of siRNA mediated knock-down of AhR on the ability of 2AI to exercise its protective effects. 2AI could no longer protect against 4HNE mediated cytotoxicity following knock-down, but this was not observed using the non-targeting siNT control (Fig. 3C). We also tested an inhibitor of AhR signaling, *a*NF (alpha-naphthoflavone) on chemotoxicity of 4HNE and action of 2AI. *a*NF has been shown to inhibit ligand dependent activation of AhR in a number cellular systems by agonists such as TCDD^29^,^30^. It has also been shown that *a*NF can directly bind to the Cyp1 active site and inhibit the activity of this enzyme^25^,^28^,^31^. Treatment with *a*NF prevents the ability of both 2AI (Fig. 3D) and TCDD (Fig. S3B) to rescue 4HNE mediated cytotoxicity. Additionally, treatment with 2AI diminished the 4HNE dependent increase in reactive oxygen species as judged by the ROS tracer dye DCFDA (Fig. 3E), suggesting that this might be a mechanism of its cytoprotective action.

The ability to derive tissue specific cells from human embryonic stem cell (hESC) cultures has revolutionized the analysis of small molecules in more disease relevant systems. We have previously described the generation of mature RPE cells from hESCs cultures^32^. Here, we used these mature hESC-RPE cells to further confirm 2AI activity. Upon 2AI treatment, the hESC-derived RPE cells have increased expression of CYP1 enzymes as expected (Fig. 3F). Additionally, treatment with 2AI was chemoprotective against 4HNE mediated cytotoxicity in these hESC-RPE cells (Fig. 3G). This shows that the AhR ligand 2AI can protect ARPE19 and hESC-derived RPE cells from 4HNE mediated cytotoxicity.

Another interesting feature of AhR activation in human RPE cells is the NRF2 cross talk which has also been shown in murine liver cells^33^,^34^. Our results indicate that activation of AhR can up regulate known downstream effectors of the anti-oxidant transcription factor NRF2^35^ (GCLM, NQO1 and HMOX1) along with previously described AhR effectors (ALDH1 and CYP1) as shown in Fig. 3H. This reveals an interesting AhR-NRF2 pathway cross-talk in RPE cells, which might be a robust stress response network in these cells as described in other systems^34^,^36^,^37^. It remains to be tested if this interaction is direct in RPE cells.

The above results show that AhR and its downstream targets play an important role in the viability of RPE cells and their protection from reactive lipid aldehydes.

### Activation of AhR by TCDD and 2AI increase levels of unsaturated fatty acids in human RPE cells

**Figure 4:**
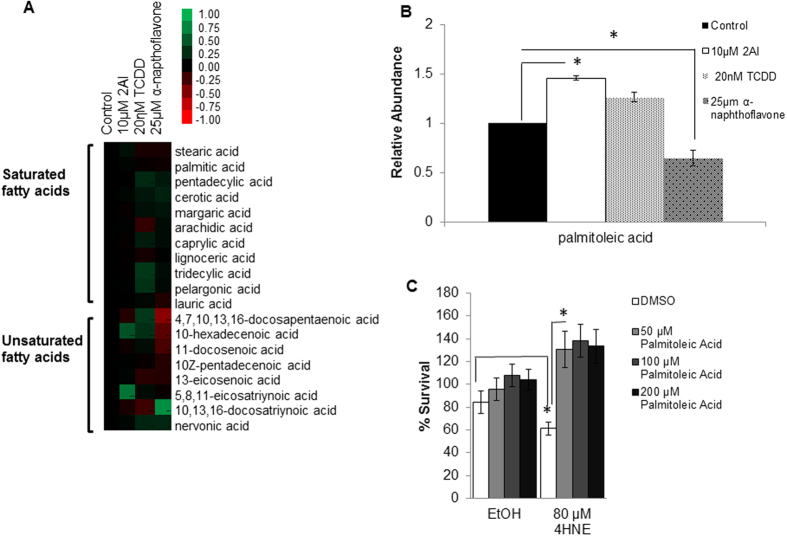
Ligands of AhR Modulate levels of intra-cellular fatty acids in human RPEs (**A**) Heat map of the relative changes in intra-cellular saturated and unsaturated fatty acids in response to treatment with AhR activators (20 nM TCDD or 10 μM 2AI) and inhibitor (25 μM aNF) for 48 hr in hESC-RPE cells. (**B**) Relative levels of intra-cellular palmitoleic acid (PA) in response to treatment with 2AI, TCDD or aNF in hESC-RPE cells. (**C**) Viability of 4HNE treated human ARPE19 cells in the presence or absence of palmitoleic acid. (n = 4, *p < 0.05).

Previous work suggests that AhR may play a role in lipid metabolism^38^,^39^,^40^,^41^. We developed an unbiased lipidomics platform for profiling RPE cells derived from hESCs treated with AhR modulators. We tested the effects of two activators of AhR- TCDD and 2AI and an AhR inhibitor aNF on intermediates of cellular metabolism. Treatment of these cells with 2AI or TCDD did not have any statistically significant changes in cell viability as judged by measurement of total cellular ATP (Fig. S3I). Figure 4A shows a heat map of the relative changes in both saturated and unsaturated lipid intermediates in human RPE cells treated for 48 hours with modulators of AhR signaling. Activation of AhR using the AhR agonist TCDD^13^ and 2AI up-regulates intra-cellular levels of (n-3) poly-unsaturated fatty acids (PUFA) and mono-unsaturated fatty acids (MUFA) lipids (3-docosapentenoic acid and hexadecatetraenoic acid, and hexadecenoic acid). Also when treated with an AhR antagonist *a*NF there is a corresponding decrease in these levels (Fig. 4A). This suggests that AhR can control unsaturated fatty acid metabolism, and up regulate beneficial lipid molecules in RPE cells.

Notably one of the MUFA lipids that is up regulated by AhR-activation is hexadecenoic acid (also called palmitoleic acid (PA)) (Fig. 4A,B). This lipid has been shown to have numerous beneficial metabolic effects in other systems^42^. Palmitoleic acid may be an important therapeutic agent for preserving RPE cells in AMD. We found a significant increase in palmitoleic acid upon treatment with 2AI as with TCDD and a decrease in levels with *a*NF treatment (Fig. 4B). This suggests that modulating levels of the mono-unsaturated fatty acid might be a down-stream effector of AhR activation. We next tested whether providing MUFA to ARPE19 cells could mediate chemoprotective effects against 4HNE toxicity. Concurrently, we found that pre-treatment of RPE cells with palmitoleic acid protected these cells from 4HNE mediated cytotoxicity in a concentration dependent fashion (Figs 4C and S3E). No protective effects were observed by using a different related saturated fatty acid (palmitic acid) control (Fig. S3G), suggesting a unique effect of palmitoleic acid. This suggests that activation of AhR might promote the intra-cellular generation of MUFA as protective intermediates. In agreement with this, 2AI (5 μM) robustly increased expression levels of the genes SCD1 and CD36 (Fig. S3H) which play an important role in modulating fatty acid metabolism in ocular and other tissues^43^,^44^,^45^,^46^.

### 2AI and palmitoleic acid protect retinal cells from light stress *in vivo*

**Figure 5:**
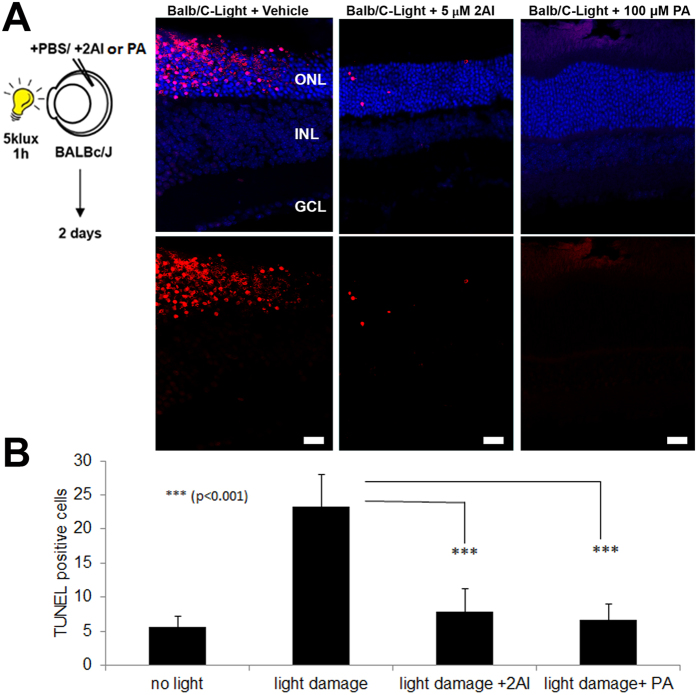
AhR ligands and MUFA effector promote retinal protection. (**A**) Retinal sections stained for TUNEL (red) to mark apoptosis showing that 5 μM 2AI and 100 μM Palmitoleic acid (PA) are protective against light-stress (5Klux for 1 hour) following intravitreal injection in the Balb/C retina compared to control (PBS-injected). Eyes were assayed at 48 hours. TUNEL positive retinal cells are quantified in (**B**) (n = 4/condition). (Scale bar = 20 μm).

Since AhR activation had a protective effect *in vitro* and is critical in retinal homeostasis, we hypothesized that ligands identified from our *in silico* screen for AhR mediated activation of the pathway may promote retinal protection against stress. To test this, we used a model of retinal damage induced by light exposure as described above (Fig. 1 and S1A). Mice of the BalB/c strain lack the protective variant of the Rpe65 allele, which renders them highly susceptible to light induced retinal damage^18^. Consistently, after exposure of these mice to 5klux of bright light for 1 hour, we observed significant induction of photoreceptor apoptosis as assayed by TUNEL (Fig. 5A). Intravitreal injection of 2AI (5 μM) one hour prior to light exposure significantly reduced apoptosis when assayed two days later (Fig. 5A middle panel and quantified in Fig. 5B). Since MUFAs are highly up-regulated downstream of AhR activation especially palmitoleic acid, we tested if it would be sufficient to protect the retina from damage in this model. Consistent with our *in vitro* data, Palmitoleic acid (100 μM) was also protective against light stress (Fig. 5A right panel and Fig. 5B). The data suggests that 2AI has protective effects on RPE cells both *in vitro* and *in vivo*, and at least some of the protective effects of the AhR activation may act through lipid effectors.

## Discussion

A key environment-sensing transcription factor is AhR, a ligand-activated Per-ARNT-Sim (PAS)/bHLH domain containing transcription factor^10^. The physiological role of this conserved transcription factor and the nature of its endogenous ligands are not well understood. The AhR knockout mouse has provided new insights into the physiological role of AhR. Recent studies using AhR knockout mice have described a retinal degeneration phenotype which is similar to that seen in AMD^8^,^9^. The knock-out of AhR in mice leads to the age-related degeneration of RPE cells, suggesting a critical role for AhR in maintaining the homeostasis in retinal cells. We show that under conditions of light-induced stress there is a clear nuclear translocation of AhR protein in both C57Bl6 and Balb/C mice (Fig. 1) validating the role of AhR as a stress-response gene in the retina. In addition, we show that AhR may cross-talk with the NRF2 pathway, thereby boosting the RPE cell stress response (Fig. 3H). It is interesting to note that the effect of AhR-activation on ROS seems to be tissue dependent. It has been reported that in lung mucus hepatocyte and breast derived can cell lines that activation of AhR increases ROS levels and oxidative stress^47^,^48^, possibly by the up regulation of NADPH oxidase. In retinal cells it has been shown AhR deficient mice have high levels of inflammation and maybe subjected to prolonged ROS stress^49^,^50^. This study suggests that in the eye activation of AhR by 2AI confers a protective role for AhR, probably by activating the AhR-NRF2 anti-oxidant battery of genes. The difference in pro and anti-oxidant effects of AhR activation could be due to the differential regulation of this battery in a tissue specific manner, though this remains to be comprehensively explored in future studies.

AhR does get down regulated under toxic light conditions especially in photoreceptors in Balb/C mice and so identifying ways to boost the pathway may promote neuroprotection. Interestingly Malek and colleagues^9^ have shown using human samples that there is an age-associated loss of AhR activity even though levels of AhR protein are unchanged. This further suggests that activating the down-stream effectors of this pathway may be a possible intervention to restore homeostasis and prevent retinal degeneration.

To test this hypothesis, we characterized a novel indole containing synthetic activator of the AhR pathway that can protect human RPE cells *in vitro* from metabolic toxicity (Figs 2 and 3). This synthetic AhR ligand, 2AI, maintains RPE cell viability during 4HNE-mediated ROS stress *in vitro* as well as photoreceptor viability during light-stress in BalB/C mice *in vivo* (Fig. 5). This protective ability is important since It has been shown that 4HNE levels are induced by light and oxidative stress in retinal cells^17^,^29^ leading to cell death. This suggests that AhR mediated protection against 4HNE might be important for coping with light induced stress in the eye and that promoting this activity using novel drugs might play an important therapeutic role in retinal neuroprotection.

An important factor that is less understood in diseases of retinal dysfunction (e.g. AMD) is the role of lipid homeostasis^39^,^40^. It is known that dietary lipids and lipid metabolism are significant modifiers of AMD. Diets rich in fish and unsaturated fatty acids have a negative correlation towards risk for AMD in population studies^41^. Our results show that AhR-ligands play an important role in regulating lipid metabolism. Treatment with 2AI up-regulates the expression of CD36 and SCD1 (Fig. S3H) which are known modulators of lipid unsaturation. Using human RPE cells, we discovered AhR regulated lipids using our Liquid Chromatography-Mass Spectrometry (LC-MS) platform. We identified an omega-7 monounsaturated fatty acid, commonly known as palmitoleic acid, as a downstream effector of AhR activation, which we also show to be protective against a lipid peroxidation product, 4HNE, in human RPE cells (Fig. 4). Finally, treatment of a murine model of retinal degeneration with palmitoleic acid can protect retina from light stress (Fig. 5). Lipid mediators might act by changing the composition of membrane lipids and thereby the properties of cell membranes. The nature and mechanisms by which mono-unsaturated lipids mediate their chemoprotective effects will be an important area of investigation in the future. It is interesting to note that palmitoleic acid has been shown to have protective effects on beta-cells and to be a liver-generated lipokine that plays a central role in maintaining metabolic homeostasis^17^. On the other hand a study has shown cytotoxic effects in hepatoma cells^29^. This suggests that the protective action of this lipid might be tissue specific.

In conclusion, this study shows that AhR plays a critical role in RPE homeostasis as a stress response pathway and by modulating lipid metabolism. Novel indole containing AhR activators and their down-stream lipid modulators can be important novel targets for a number of retinal degenerations including AMD.

## Materials and Methods

### Chemicals

Palmitic acid, indole-3-carbinole (I3C), 2-(2-aminophenyl) indole (2AI), *a*-napthoflavone (*a*NF), ammonium acetate, and DMSO were obtained from Sigma Aldrich (St. Louis, MO). Palmitoleic acid and 4-Hydroxy Nonenal (4HNE) was purchased from Cayman Chemical Company (Ann Arbor, MI, USA). 2,3,7,8-Tetrachlorodibenzo-P-Dioxin (TCDD) was acquired from Cambridge Isotope Laboratories, Inc. (Tewksbury, MA, USA). HPLC-grade solvents acetonitrile and methanol were purchased from Fisher Scientific (Pittsburgh, PA, USA) and VWR (Radnor, PA, USA). Deionized water was generated in-house for mobile phase preparation.

### Lipidomics

For high-resolution (accurate-mass) HPLC-MS analysis, HPLC was performed using a Agilent 1260 Infinity LC system fitted with following modules: u-degasser (G1322A), binary pump (G1312B), thermostated column compartment (G1330B), and HiPALS auto sampler (G1367E). Chromatographic separation of intracellular lipid extracts was performed on a Phenomenex Luna NH_2_ (2.0 mm × 150 mm, 3.0 μM) column. The solvent system was A = 20 mM ammonium acetate pH 9.5 with 5% acetonitrile and B = acetonitrile. The starting gradient conditions were 95% B at a flow rate of 0.3 mL/min. The following gradient program was used: 0 to 20 min, 95–10%B, 25–30 min 10%B, and 30.1–35 min 95%B. Samples were kept at +4 °C, and the injection volume was 10 μL.

High-resolution MS1 was performed using an Agilent 6520 QTOF mass spectrometer fitted with a Dual-Spray Electrospray Source (ESI). The instrument was operated at a mass resolution of ~20,000 for TOF MS1 scan using 2GHx extended dynamic range mode. The ionization parameters were set as follows: gas temperature(TEM) 350 °C; drying gas, 9 L/min; Vcap, 2500 V; nebulizer, 35 psig; fragmentor, 125 V; and skimmer, 65 V. MS1 acquisition was operated in the negative ion scanning mode for a mass range of 50–1500 m/z.

The HPLC-MS data was acquired using Agilent MassHunter Workstation (B.05.00). Agilent MassHunter Qualitative Analysis Software (B.07.00), Mass Profiler Professional (B.12.0), and Microsoft excel 2007 (Redmond, WA, USA) were used for an in-depth MS1 analysis of cellular extracts. To perform a comparative quantitation of metabolites, peak area were assigned using Agilent MassHunter Qualitative Analysis Software in combination with the Find by Formula (FBF) algorithm. Peak areas were normalized by total protein.

### Light Exposure of Mouse Retinas

All mice used in this study were housed and bred at the AAALAC accredited vivarium of The Buck Institute for Research on Aging in a Specific Pathogen Free (SPF) facility and housed in individually ventilated cages on a standard 12:12 light cycle. All procedures were approved by the Buck Institute Institutional Animal Care and Use (IACUC) Committee and carried out in accordance with the approved guidelines.

BALB/cJ albino (JAX, stock nr. 000651) and C57BL/6J pigmented (JAX, stock nr. 000664) wild type mice were purchased from The Jackson Laboratories (JAX). Animals were anesthetized for 15 min in an isoflurane chamber and pupils were dilated using a mixture of 5% Phenylephrine (Arcos Organics) and 1% Tropicamide (Alfa Aesar). Mice were kept under anesthesia during light exposure to ensure direct and constant illumination during the exposure time. Light intensity at the site of exposure was measured using a digital lux meter (LX1330B, Sinometer). Mice were dark adapted for 18 h before the procedure. Test eyes were exposed to 5,000 lux (BALB/cJ) or 8,000 lux (C57BL/6J) of bright light using a 144-LED microscope ring light (AmScope) for 1 hour (BALB/cJ) or 1.5 hours (C57BL/6J). After light damage, mice were allowed to recover from anesthesia, returned to their cages and housed in darkness until analysis. Undamaged control mice were housed in regular conditions (see above) throughout the experiment.

### Mouse Retinal Histology and Immunohistochemistry

Mouse eyes were harvested and fixed in 4% paraformaldehyde for 1 hour at 4 °C and transferred to 1× PBS until processing (24-hour maximum storage time). Further fixation of the harvested eyes took place in 15% sucrose in 1x PBS for 1 hour. Fixed eyes were then embedded in 7.5% gelatin solution with 15% sucrose in 1x PBS. Once the embedding media is solidified, eyes were snap-frozen in pre-chilled methylbutane on dry ice and stored at −80 °C until cryosectioning.

For immunohistochemistry, sections were rinsed with 1× PBS twice for 5 minutes each and then incubated for 30 minutes at room temperature with 5% fetal bovine serum (Atlanta Biologicals) and 1% Triton-X100 (Sigma-Aldrich) in 1× PBS. Primary antibodies for mouse AhR (Abnova) at a dilution of 1:100 and CYP1A (LSBio) at a dilution of 1:200 were then applied to the sections for overnight incubation at 4 °C. After washing the sections with 1× PBS twice for 5 minutes each, relevant secondary antibodies were conjugated with Alexa Fluor 488 or 594 (Life Technologies) and then applied to the sections for 1 hour at 4 °C. After rinsing with 1x PBS twice for 5 minutes each, coverslips were applied to the sections with ProLong Gold antifade reagent with DAPI (Life Technologies) and sealed with nail polish. TUNEL staining was done according to the manufacturer’s recommended instructions (R&D Systems Sections were imaged using a Zeiss LSM 700 confocal microscope (Carl Zeiss Meditec, Jena, Germany).

### Cell Culture

ARPE19 cells were purchased from American Type Culture Collection (ATCC) and maintained in Dulbecco’s Modified Eagle Medium (Corning) with 10% fetal bovine serum (Atlanta Biologicals) and 1% Penicillin-Streptomycin (Life Technologies). Cells were grown in a 37 °C incubator with 5% CO_2_ and media was changed every other day.

For deriving retinal pigmented epithelial cells from pluripotent stem cells, undifferentiated embryonic stem cells were maintained in Essential 8 basal medium (Life Technologies) supplemented with 1% Essential 8 supplement (Life Technologies) and 1% Penicillin-Streptomycin-Amphotericin B (Lonza) on culture vessels coated with Matrigel (BD Biosciences). Cells were grown in a 37 °C incubator with 5% CO_2_ and 5% O_2_. Media was changed every day.

For the differentiation of embryonic stem cells into the RPE cell fate, pluripotent stem cells were subjected to a 3-week treatment with 10 ng/mL DKK1 (R&D Systems), noggin (R&D Systems), and IGF1 (R&D Systems) in Dulbecco’s Modified Eagle Medium/F-12 1:1 with 2.50 mM L-glutamine and 15 mM HEPES (HyClone), 1% sodium pyruvate (Corning), 1% N-2 Supplement (Life Technologies), 1% HEPES (HyClone), and 1% Penicillin-Streptomycin-Amphotericin B (Lonza). Spontaneously-appearing pigmented foci were manually picked and transferred to a separate culture vessel coated with Matrigel for expansion and maturation. Differentiated RPE cells were maintained in MEM/EBSS with 2.00 mM L-glutamine and Earle’s balanced salts (Hyclone) supplemented with Knockout^tm^ serum replacement (Life Technologies), 1% for maintenance of mature RPE cells and 5% for the proliferation of RPE cells to 100% confluency before maturation, 1% Penicillin-Streptomycin-Amphotericin B (Lonza), 1% Glutamax (Life Technologies), 125 mg taurine, 10 μg hydrocortisone, and 6.5 ng triiodo-thyronin. Cells were maintained in a 37 °C incubator with 5% CO_2_ and the media was changed every other day.

### AhR ligand competition assay

Binding experiments were performed using 2-azido-3-[125I]iodo-7,8-dibromodibenzo-p-dioxin and hepatic cytosol from B6.Cg-*Ahr*^*tm3.1Bra*^ Tg (Alb-cre, Ttr-AhR)1GHP humanized mice as previously^25^,^26^ described. All binding experiments were conducted in the dark until UV-mediated activation of the AHR photoaffinity ligand 2-azido-3-[125I]iodo-7,8-dibromodibenzo-p-dioxin (PAL) as described previously^25^. In brief, ligand-treated lysates were incubated at room temperature for 20 min and then photolyzed at 8 cm with 402 nm UV light. Dextran-coated charcoal (1%) was added to the photolyzed samples, which were then centrifuged at 3000 g for 10 min to remove free ligand. Labeled samples were resolved using 8% acrylamide-tricine SDS-PAGE, transferred to PVDF membrane, and visualized using autoradiography. Labeled AHR bands were excised and counted using a γ counter. A saturating amount of the PAL (0.21 pmol, 8 × 10^5^ cpm per tube) was added to 150 μg of total protein of mouse liver along with increasing amounts of competing ligands BNF and 2AI.

### siRNA Treatment

The control small interfering RNA (siRNA) pool of non-targeting sequences and siRNA pool for mouse AhR (GE Dharmacon) were transfected into cells using Dharmafect 1 (GE Dharmacon). ARPE19 cells were plated in a 6-well format with a seeding density of 100,000 cells/well 24 hours before transfection. 1 hour prior to transfection, cell culture maintenance media was removed and replaced with 1× Opti-MEM reduced serum media (Life Technologies). Cells were then transfected with 5 μM of either the non-targeting siRNA pool or mouse siAhR pool for 24 hours. After which, the Opti-MEM/Dharmafect/siRNA mixture was removed and replaced with cell culture maintenance media. After 48 hours, RNA was extracted for real-time quantitative PCR analysis.

### Real-Time qPCR Analysis

Total RNA was extracted from cells using the Quick-RNA MiniPrep kit (ZymoResearch) according to the manufacturer’s recommended instructions. RNA samples were then quantified by a NanoDrop 2000 apparatus (Thermo Scientific). cDNA was synthesized from 0.25–0.5 μg of total RNA template using the iScript 5x RT Supermix (Bio-Rad). Reactions occurred in a T100 Thermal Cycler (Bio-Rad) according to the manufacturer’s recommended instructions. Real-Time Quantitative PCR analysis was performed using a Bio-Rad CFX Connect Real-Time PCR Detection System. Acquisition of data was then performed on a Bio-Rad CFX Manager software. Each PCR reaction comprised of both forward and reverse primers at a concentration of 400 ηM with 1 μL of cDNA diluted five-fold, 7.4 μL of nuclease-free water, and 10 μL of Sso Advanced Universal SYBR Green Supermix (Bio-Rad). Sample data values from quantitative PCR analysis were normalized to TBP (Table 1).

### Viability Assays

To determine the role of AhR signaling in maintaining cell viability in light of 4HNE-mediated cytotoxicity, cells were plated in a 96-well format with a density of 150,000 cells per plate. Cells were then treated with desired concentrations of 2,3,7,8-tetrachlorodibenzo-p-dioxin (Cambridge Isotope Laboratories), 2-(2-aminophenyl) (Sigma-Aldrich), palmitoleic acid (Cayman Chemical Company) or control DMSO (Sigma-Aldrich). In determining viability dependence on AhR, cells were co-treated with AhR ligands and desired concentrations of α-naphthoflavone (Sigma-Aldrich). Molecule treatments lasted for 48 hours and were and co-treatment with 4-hydroxynonenal (Cayman Chemical Company) for 24 hours. At which point, cell viability was assessed using the CellTiter-Glo luminescent cell viability kit (Promega. Inc) and a Fluoroskan Ascent FL luminometer. Cell viability was also accessed using Calcein-AM kit (Life. Inc.) and a Spectramax fluorescence plate reader.

### Dichlorofluorescein Assay for ROS

To assess abundance of cytoplasmic ROS, 2′,7′-dichlorofluorescein 3′,6′-diacetate (Acros Organics) was incubated with cells in 1x Hank’s Balanced Salt Solution (Cellgro) at a working concentration of 5 μM for 30 minutes before images were taken using epifluorescence microscopy.

### Intraocular Injections in mice

Mice were anesthetized using isoflurane inhalation. Following confirmation of the depth of anesthesia, 2AI, PA or PBS (control) in 1 μl volume were injected into the right eye using a graduated pulled glass pipet and a wire plunger (Wiretrol II, 5-0000-2005, Drummond Scientific Company). The pipet was used to poke a hole just beyond the corneo-scleral margin, further advanced carefully, and the solution delivered into the vitreous cavity. The pipet was kept in place for 30 secs to allow the intra-ocular pressure to normalize and then gently withdrawn.

### TUNEL staining of mouse retinal cryosections and Quantification

Cryosections on slides were thawed and hydrated in PBS for 15 minutes at room temperature (RT). Tissue was permeabilized and washed 3 times with PBS. Cryosections were incubated in the dark for 1 h at 37 °C with 50 ul of TUNEL reaction mixture (*In situ* cell death detection kit, TMR Red, Roche) prepared according to manufacturer’s instructions. Slides were rinsed three times with PBS, counterstained with DAPI and mounted for image acquisition. TUNEL + nuclei were counted in 1024 × 1024px fields from confocal captures of retinal sections stained with TUNEL and DAPI. Quantifications include 5–6 independent sections per eye, covering 2/3 of the retina, in a total of at least 3–5 eyes per condition.

### *In vitro* light-stress on RPE cells

hESC-RPE cells were cultured in 12-well culture plates. Up to three wells were exposed to a LED light source (ESCO-Lite) emitting 400 nm (+/−10 nm) light for 30–60 minutes. The cells were then further cultured for 12 hours in the dark prior to mRNA extraction.

### Statistical Analysis

All quantification are presented as average and standard error of mean (s.e.m.). Statistical analysis was carried out using Microsoft Excel or GraphPad Prism and student’s t-test was used to determine statistical significance, assuming normal distribution and equal variance.

## Additional Information

**How to cite this article**: Gutierrez, Mark A. *et al*. A novel AhR ligand, 2AI, protects the retina from environmental stress. *Sci. Rep.* **6**, 29025; doi: 10.1038/srep29025 (2016).

## Supplementary Material

Supplementary Information

## Figures and Tables

**Table 1:** PCR primer sequences.

Gene	Forward Sequence	Reverse Sequence
TBP	cggctgtttaacttcgcttc	cacacgccaagaaacagtga
AhR	agtggtcccagcctacacc	cgactggcgtaggtgatgt
CYP1a1	ggagcactacaaaacctttgaga	tcatctgacagctggacattg
CYP1b1	tgagtgccgtgtgtttcgg	gtgcctcaagaacttgtccag
CD36	tggaacagaggctgacaactt	ttgattttgatagatatgggatg
NRF2	atgacaatgaggtttcttcgg	caatgaagactgggctctc
KEAP1	gagaatctacgtccttggagg	caggtgtctgtatctgggtc
GCLC	aagtggatgtggacaccag	ctgtcattagttctccagatgc
GCLM	gttgacatggcctgttcag	aactccatcttcaataggaggt
NQO1	acatcacaggtaaactgaagg	tcagatggccttctttataagc
HMOX1	aactccctggagatgactc	ctcaaagagctggatgttgag
SCD1	cctagaagctgaggaactggtg	acatcatcagcaagccaggt

Sequences of PCR primers used to measure expression of genes related to the AhR and NRF2 pathways are listed in the table.
