# Supplementary material for: A novel AhR ligand, 2AI, protects the retina from environmental stress
Source: Sci Rep. 2016 Jul 1;6:29025. doi: 10.1038/srep29025 (PMC4929558; doi:10.1038/srep29025)
Supplement: Supplementary Information [file srep29025-s1.pdf]

Supplementary Materials

A novel AhR ligand, 2AI, protects the retina from environmental stress.

Gutierrez Mark A. [1], Davis Sonnet S. [2], Rosko Andrew[2], Nguyen Steven M. [2], Mitchell Kylie P. [2], Mateen Samiha[2], Neves Joana[2], Garcia Thelma[2], Mooney Shawn [3], Perdew Gary H [4], Hubbard, Troy D.[4], Lamba Deepak A [2*] and Ramanathan Arvind [2 *]

Methods

In silico homology modeling and small molecule docking and model validation

We used the program SCWRL [1] to build side chains on the backbone template of 3F1O. Next we assessed whether and how to minimize the energy of the model generated by SCWRL. Using the set of known ligands [2] (Table 1), we determined the correlation between predicted and measured binding affinity for the structure directly from SCWRL [1], which was 0.35. After energy minimization with AMBER in Chimera[3], this decreased to 0.26. This shows that without including a bound ligand, energy minimization is counterproductive to model performance for docking. Although docking the known high-affinity agonist TCDD to the un-minimized structure gave a relatively low score, minimization of this complex using the same procedure as above gave a structure that bound TCDD well and increased the affinity correlation to 0.71. Since, we were primarily interested in predicting binding affinities of small molecules, specifically indole derivatives, we chose to use this last model.

We evaluated our models using a set of ligands from TableS1 [2] , which includes TCDD and a number of indole compounds, as well as kynurenine and kynurenic acid, two previously reported endogenous AhR activators from the human tryptophan catabolic pathway. These compounds span a large experimentally derived Kd range (from 7pM to 27μM) and are enriched in indole and indole-like scaffolds.

Ligands for model validation were built by importing the structures into Chimera from the PubChem database, adjusting the protonation state of ionizable groups manually, and then minimizing the energy for 100 steps within Chimera using the

generalized AMBER force field (GAFF)[4].

Autodock Vina [5,6] was used to perform flexible docking (ligand bond torsions were flexible while the protein was treated as rigid) and estimate Kds for each of the ligands. The protein homology model and ligands were prepared by assigning charges and atom types using the AutoDock Tools, and the ligands were docked into a search box that included the entire buried cavity of the protein. All bonds except ring bonds and amide C-N bonds were treated as rotatable. The score reported by AutoDock was used to rank the resulting conformations.

In silico chemical screening

We filtered commercially available compounds from Sigma Aldrih using the following rule, in addition to containing an indole ring, we required that our compounds have a molecular weight ≤300 Da, a charge of -1 to +1, and contain only "benign" functional groups (for a list of excluded functional groups, see the "yuck rules" at http://blaster.docking.org/filtering/). These rules were chosen to provide a drug-like virtual screening library. Result compounds were downloaded in bulk from ZINC as a SDF file, which contains multiple separate pre-built 3D structures in Mol2 format. Virtual screening of this set using Vina was automated using the interface PyRx[7].

AhR luciferase reporter assay

To assess luciferase expression associated with XRE sequence transcription by treatment with our AhR ligands, we utilized HepG2 human hepatocellular carcinoma cells (ATCC). Cells were maintained in Dulbecco’s Modification of Eagle’s Medium

(DMEM; Corning) with 10% fetal bovine serum (Atlanta Biologicals) and 100 U/mL Pen Strep (Life Technologies) prior to transfection with the reporter construct. For the assay, cells were plated in a 6-well plate (Corning) with a seeding density of 200,000 cells/well. The cells were transfected the next day with the reporter construct (as described in SA biosciences CCS-2045L) using Lipofectamine 2000 (Life Technologies) with a DNA:reagent ratio of 2:3 for each well. The day after transfection, cells were treated for 48 hours with AhR ligands of interest as well as a control concentration of DMSO. After treatment, protein lysates were prepared and analyzed for luciferase abundance by a Fluoroskan Ascent FL luminometer according to the recommended protocol supplied by the Luciferase Assay System (Promega).

Results

In silico homology modeling of the AhR PAS domain can be used to predict binding of potential indole containing ligands

An important approach for evaluating the ligands of AhR has been the computational structural analysis of the ligand binding PAS domain, and ligand docking. In the absence of known crystallographic three dimensional protein structures and high resolution NMR structures, comparative modeling approaches using sequence similar templates of known structure have proven useful for the characterization of binding mode and free energy of small molecule ligands. There have been several previous studies that comparatively model the three dimensional atomic structure of the AhR protein and predict ligand binding using structure-based models [8]. With the goal of developing structure-based predictive toxicology models in different species based on

AhR sequence, Pandini *et. al.* [8] used a homology model to identify the binding mode for TCDD, and to investigate the contributions of the various side chains in the binding pocket to TCDD affinity. Bisson *et. al.* [9] used their own independently developed homology model to dock two putative endogenous ligands, and to screen a library of natural products to identify two previously unreported flavonoid agonists . Finally, Motto *et. al.* [10] were the first to propose the use of ligand-bound template structures as a means of generating more binding-relevant models, which allowed them to predict the relative affinities of TCDD analogs with the chlorine substituents at various ring positions, i.e. a rather subtle variation in structure. Following the lead of these studies, we developed a preliminary homology model of the ligand binding domain of AhR. We used the structure of the heterodimer of Hif-2α and ARNT domains bound to an artificial ligand (PDB accession code 3F1O) in an effort to capture the ligand bound conformation. The template target alignment was identical to that used by Pandini *et. al.* [8] and has a sequence identity of 27% over the 109 amino acids of the AhR PAS-B domain.

A graph of docking score (that correlates with binding energy) vs. log of experimental Kd (from published studies [2]), is shown in Fig. S2A and Table S1. The docking score was generated for each of the compounds as described in the methods section. The graph in Fig. 1A shows that the calculated docking scores correlate well (R=0.7) with the reported experimentally calculated Kd's. This supports the use of the *in silico* model for predicting new indole containing ligands. The ligands from Table S1 have a docking score of less than -6, with the most potent ligand (TCDD) having a score of -10.3.

In silico screening identified the novel indole containing ligands of AhR, 2AI

Previous studies have successfully screened for novel flavone containing ligands of AhR using similar *in silico* approaches [9]. To our knowledge this has not been performed for indole-containing small molecule libraries. We chose to screen 70 indole-containing building blocks for organic synthesis provided by Sigma Aldrich Inc.. We performed an *in silico* screen of these compounds as described in the methods section. Among the compounds from Sigma Aldrich Inc., we chose the top three compounds as hits. All of our hits were verified to have docking scores of -9 or lower, which corresponds to a Kd of 5 nM by our correlation plot (Fig S2). 2AI was the top hit in the screen which was chosen for further analysis in cellular studies.

Supplementary Table S1.

	Vina Score	log(actual Kd)
TCDD	-9.6	-11.1
ICZ	-10.3	-9.7
DIM	-9.6	-7.0
I3A	-6.7	-5.3
I3C	-6.8	-4.6
KYN	-7.3	-5.7
KYNA	-7.4	-7.0

Compound	Ctrl area (avg)	10nM TCDD area (avg)	25μM A-Naphthoflavon area (avg)	Ctrl area (stdev)	10ηM TCDD area (stdev)	25μM A-Naphthoflavon area (stdev)

Supplementary Table S2.

stearic acid	9.78E+06	8.89E+06	8.88E+06	2.06E+06	1.54E+06	1.97E+06
palmitic acid	8.95E+06	8.69E+06	8.54E+06	1.69E+06	4.47E+05	1.47E+06
pentadecylic acid	7.04E+05	8.58E+05	8.03E+05	1.13E+05	9.27E+04	1.01E+05
cerotic acid	3.70E+04	4.18E+04	4.40E+04	6.87E+03	1.03E+04	1.58E+04
margaric acid	7.04E+07	7.65E+07	7.87E+07	3.06E+06	4.56E+06	1.30E+06
arachidic acid	4.06E+05	3.31E+05	4.34E+05	7.44E+04	9.58E+04	1.07E+05
caprylic acid	2.41E+06	2.80E+06	2.57E+06	1.49E+05	1.34E+05	1.21E+05
lignoceric acid	9.75E+04	9.00E+04	9.91E+04	1.64E+04	1.39E+04	1.41E+04
tridecylic acid	4.05E+05	5.31E+05	4.33E+05	4.66E+04	3.80E+03	5.99E+04
pelargonic acid	7.92E+05	1.01E+06	8.33E+05	5.10E+04	5.91E+04	1.77E+05
lauric acid	1.08E+06	1.13E+06	9.49E+05	2.70E+05	1.68E+05	8.69E+04
4,7,10,13,16-docosapentaenoic acid	7.78E+05	1.02E+06	3.69E+05	3.35E+05	4.19E+05	1.25E+05
10-hexadecenoic acid	2.46E+05	3.11E+05	1.59E+05	2.70E+04	4.94E+04	4.97E+03
11-docosenoic acid	1.07E+05	1.14E+05	7.72E+04	1.71E+04	1.24E+04	2.39E+03
10Z-pentadecenoic acid	2.00E+05	1.95E+05	1.73E+05	3.64E+04	6.37E+04	5.62E+04
13-eicosenoic acid	3.56E+05	3.00E+05	3.08E+05	1.52E+05	2.27E+04	5.31E+04
5,8,11-eicosatriynoic	6.07E+0	6.45E+06	5.83E+06	9.85E+0	8.70E+04	1.41E+05

acid	6			5		
10,13,16-docosatriynoic acid	5.31E+04	4.09E+04	9.55E+04	2.20E+04	3.00E+03	7.68E+03
nervonic acid	5.69E+04	6.85E+04	6.87E+04	9.68E+03	9.97E+03	7.16E+03

**Compound**	**Ctrl area (avg)**	**5 μM 2AI area (avg)**	**Ctrl area (stdev)**	**5 μM 2AI area (stdev)**

Supplementary Table S3.

stearic acid	1.03E+07	1.12E+07	1.87E+06	1.40E+06
palmitic acid	1.29E+07	1.33E+07	7.07E+05	3.46E+05
pentadecylic acid	1.34E+06	1.34E+06	5.68E+03	3.94E+03
cerotic acid	1.43E+05	1.48E+05	1.47E+04	9.47E+03
margaric acid	5.26E+06	5.17E+06	3.52E+05	2.52E+05
arachidic acid	3.24E+05	3.30E+05	3.91E+04	2.24E+04
caprylic acid	1.20E+06	1.18E+06	1.68E+05	1.03E+05
lignoceric acid	2.25E+05	2.23E+05	1.46E+04	6.01E+03
tridecylic acid	2.78E+05	2.76E+05	2.64E+04	1.91E+04
pelargonic acid	8.63E+05	8.76E+05	7.83E+05	7.79E+05
lauric acid	5.60E+05	5.61E+05	2.46E+05	2.47E+05
4,7,10,13,16-docosapentaenoic acid	3.39E+04	2.95E+04	8.41E+03	6.44E+03
10-hexadecenoic acid	5.80E+06	8.46E+06	2.24E+06	3.10E+06
11-docosenoic acid	5.23E+04	4.97E+04	5.87E+03	3.10E+03
10Z-pentadecenoic acid	2.08E+05	2.12E+05	2.13E+03	5.74E+03
13-eicosenoic acid	3.33E+05	3.41E+05	2.05E+04	7.44E+03
5,8,11-eicosatriynoic acid	2.12E+05	3.49E+05	2.72E+05	2.77E+05
10,13,16-docosatriynoic acid	8.42E+04	7.62E+04	2.05E+04	2.18E+04
nervonic acid	3.32E+04	3.46E+04	2.60E+03	4.99E+03

References

1 Krivov, G. G., Shapovalov, M. V. & Dunbrack, R. L., Jr. Improved prediction of protein side-chain conformations with SCWRL4. *Proteins* **77**, 778-795, (2009).

2 Bjeldanes, L. F., Kim, J. Y., Grose, K. R., Bartholomew, J. C. & Bradfield, C. A. Aromatic hydrocarbon responsiveness-receptor agonists generated from indole-3-carbinol in vitro and in vivo: comparisons with 2,3,7,8-tetrachlorodibenzo-p-dioxin. *Proc Natl Acad Sci U S A* **88**, 9543-9547, (1991).

3 Pettersen, E. F. *et al.* UCSF Chimera--a visualization system for exploratory research and analysis. *J Comput Chem* **25**, 1605-1612, (2004).

4 Wang, J., Wolf, R. M., Caldwell, J. W., Kollman, P. A. & Case, D. A. Development and testing of a general amber force field. *J Comput Chem* **25**, 1157-1174, (2004).

5 Seeliger, D. & de Groot, B. L. Ligand docking and binding site analysis with PyMOL and Autodock/Vina. *J Comput Aided Mol Des* **24**, 417-422, (2010).

6 Trott, O. & Olson, A. J. AutoDock Vina: improving the speed and accuracy of docking with a new scoring function, efficient optimization, and multithreading. *J Comput Chem* **31**, 455-461, (2010).

7 Dallakyan, S. & Olson, A. J. Small-Molecule Library Screening by Docking with PyRx. *Methods Mol Biol* **1263**, 243-250, (2015).

8 Pandini, A., Denison, M. S., Song, Y., Soshilov, A. A. & Bonati, L. Structural and functional characterization of the aryl hydrocarbon receptor ligand binding domain by homology modeling and mutational analysis. *Biochemistry* **46**, 696-708, (2007).

9 Bisson, W. H. *et al.* Modeling of the aryl hydrocarbon receptor (AhR) ligand binding domain and its utility in virtual ligand screening to predict new AhR ligands. *J Med Chem* **52**, 5635-5641, (2009).

10 Motto, I., Bordogna, A., Soshilov, A. A., Denison, M. S. & Bonati, L. New aryl hydrocarbon receptor homology model targeted to improve docking reliability. *J Chem Inf Model* **51**, 2868-2881, (2011).

Figure Legends

Supplementary Figure S1: (A) Light stress leads to photoreceptor death in Balb/C mice. Representative image showing TUNEL labeling (red) in the photoreceptor layer in mice 24 hours following 5Klux light exposure. (B) qPCR analysis comparing AhR expression in neural retina of Balb/C mice before and 24 hours following light exposure. (n=3, * $p<0.05$).

Supplementary Figure S2- Workflow of in silico screening for novel indole ligands of AhR. (A) A homology model of the ligand binding PAS domain of AhR was constructed and validated with known ligands of AhR, yielding a correlation between predicted binding (Vina score on Y axis) and log of experimentally determined Kd (on X axis) of 0.7. **(B)** commercially available indole containing compounds were filtered according to chemical properties that would yield suitable drug candidate ligands of AhR

to yield 70 candidates. **(C)** 5 compounds with highest predicted affinities were identified and clustered according to their chemical substructures to identify subsets of compounds for follow-up *in vitro* cell culture studies. 2AI was followed up as a compound that was biologically active in RPE cells for follow-up.

Supplementary Fig S3. Characterization of 2AI and palmitic acid control on RPE cells. (A) siRNA mediated knockdown of AhR in human RPE cells and relative mRNA quantification of AhR and AhR targets- CYP1a1 and CYP1b1. **(B)** Viability of 4HNE treated ARPE19 cells treated with TCDD in presence or absence of the AhR antagonist *a*NF **(C)** mRNA levels of CYP1a1 following treatment with 2AI or co-treatment with varying concentrations of AhR antagonist CH223191. **(D)** % survival of ARPE19 cells treated with varying concentrations of 4HNE measured using Calcein-AM fluorescence assay. **(E)** % survival of ARPE19 cells treated with 5 μM 2AI and 100 μM PA with our without 40μM 4HNE measured using Calcein-AM fluorescence assay. **(F)** % dead ARPE19 cells treated with 5 μM 2AI and 100 μM PA with our without 40μM 4HNE measured using Ethidium Bromide fluorescence. **(G)** Effect of control palmitic acid on 4HNE mediated cytotoxicity in ARPE19 cells. **(H)** Relative mRNA quantitation of genes involved in fatty acid import (CD36) and mono-unsaturation (SCD1) in hESC derived RPE cells treated with 5 μM 2AI. **(I)** % survival of hESC derived RPE cells treated with DMSO, 20 nM TCDD and 5 μM 2AI. (n=3, * $p<0.05$; n.s – non significant).

Supplementary Figure S4- Extracted ion chromatogram of palmitoleic acid from H1-ESC RPE untreated and treated with 10uM 2AI and 1ug/mL palmitoleic acid standard at retention time 8.7 min.

Table Legends

Supplementary Table S1. List experimentally calculated Kds for ligands with their docking Vina score calculated from *in silico* docking studies with the model of the AhR PAS domain. AhR ligands that were used in docking experiments are the following- TCDD (tetrachloro dibenzo-p-dioxin), ICZ (indole 3,2 carbazole), DIM (diindolyl methane), I3A (indole 3-carbaldehyde), I3C (indole 3-carbinol), KYN (kynurenine) and KYNA (kynurenic acid)

Supplementary Table S2. Saturated and unsaturated fatty acids profiled in hESC RPE untreated and treated with 10nM TCDD and 25µM α-Naphthoflavone. Total mass spectral counts corresponding to each of the lipids along with respective errors (standard deviations, n=3) are listed. The internal standard was margaric acid. Average total ion current of the control were used to normalize relative metabolite levels. Normalization factors were 1, 0.85 and 0.90 respectively.

Supplementary Table S3. Saturated and unsaturated fatty acids profiled in H1-ESC RPE untreated and treated with 5µM 2AI. Total mass spectral counts corresponding to each of the lipids along with respective errors (standard deviations, n=3) are listed. The internal standard was margaric acid. Average total ion current of the

control were used to normalize relative metabolite levels. Normalization factors were 1 and 1.04 respectively.

Figure S1

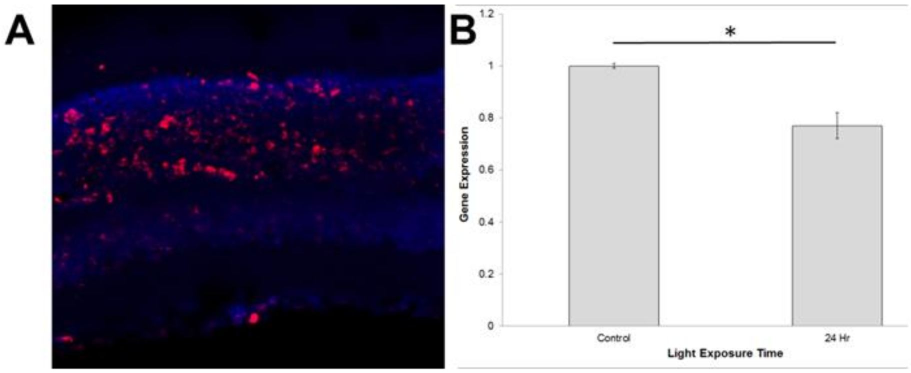
A
B
1.2
1
0.8
0.6
0.4
0.2
0
*
Gene Expression
Control
24 Hr
Light Exposure Time

Figure S2

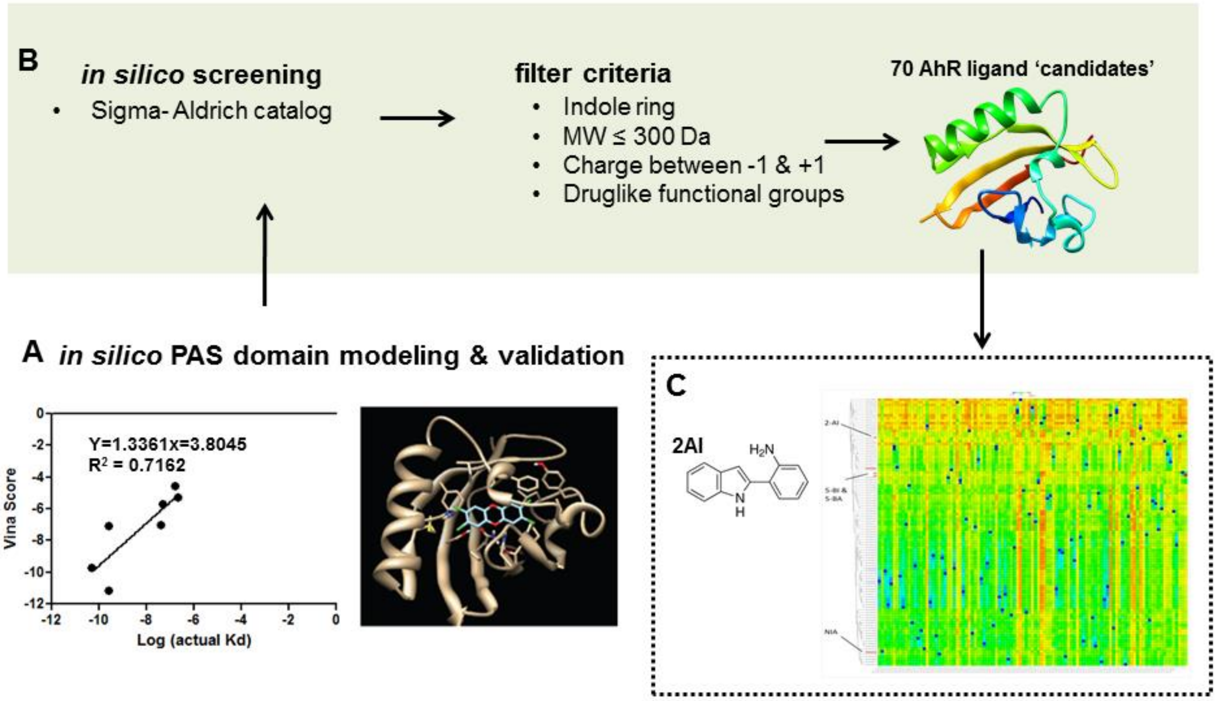

B
in silico screening
• Sigma- Aldrich catalog
filter criteria
• Indole ring
• MW ≤ 300 Da
• Charge between -1 & +1
• Druglike functional groups
70 AhR ligand 'candidates'
A in silico PAS domain modeling & validation
Y=1.3361x=3.8045
R2 = 0.7162
Vina Score
Log (actual Kd)
0
-2
-4
-6
-8
-10
-12
C
2AI
H2N
N
H

Figure S3

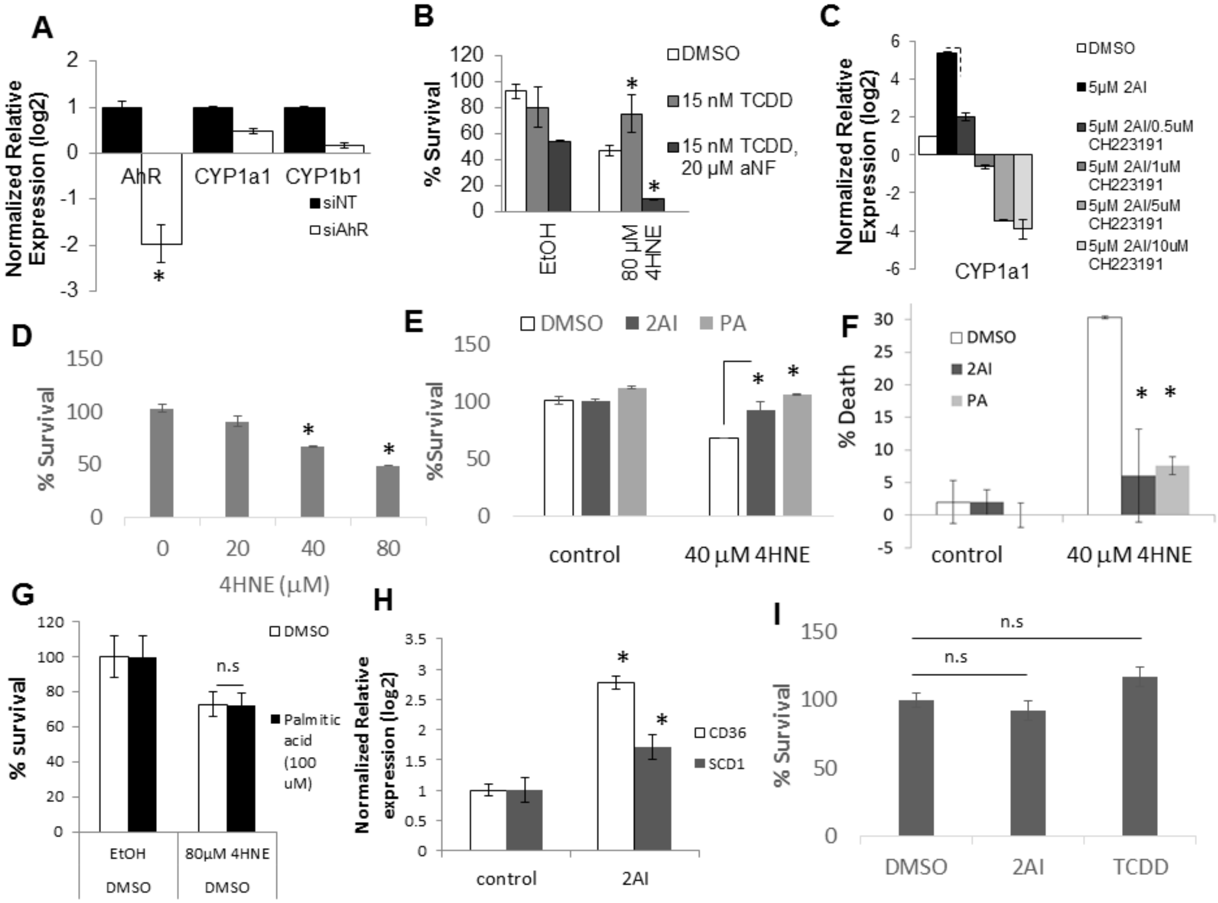

A
Normalized Relative Expression (log2)
AhR
CYP1a1
CYP1b1
siNT
siAhR
B
% Survival
DMSO
15 nM TCDD
15 nM TCDD, 20 µM aNF
EtOH
80 µM 4HNE
C
Normalized Relative Expression (log2)
CYP1a1
DMSO
5µM 2AI
5µM 2AI/0.5uM CH223191
5µM 2AI/1uM CH223191
5µM 2AI/5uM CH223191
5µM 2AI/10uM CH223191
D
% Survival
4HNE (µM)
E
DMSO
2AI
PA
%Survival
control
40 µM 4HNE
F
% Death
DMSO
2AI
PA
control
40 µM 4HNE
G
% survival
DMSO
Palmitic acid (100 uM)
n.s
EtOH
80µM 4HNE
DMSO
DMSO
H
Normalized Relative expression (log2)
CD36
SCD1
control
2AI
I
% Survival
n.s
n.s
DMSO
2AI
TCDD

Figure S4

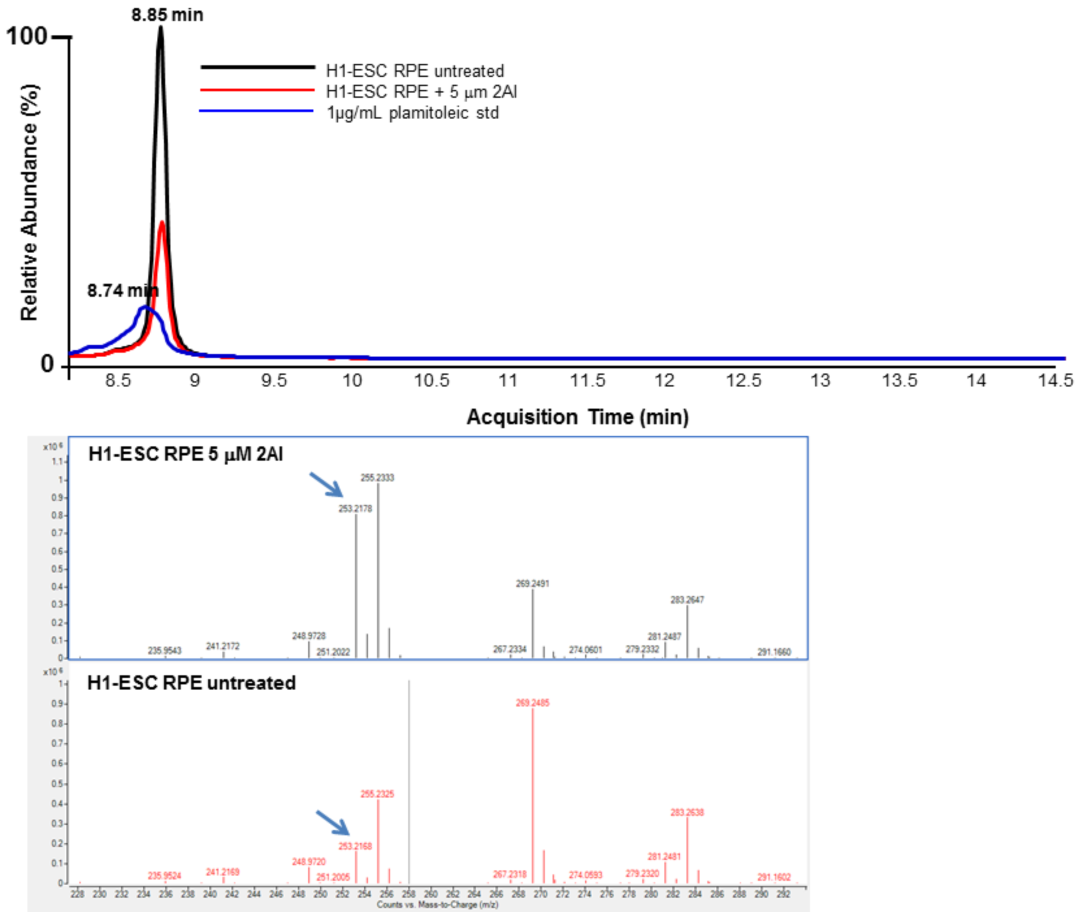
8.85 min
100
Relative Abundance (%)
H1-ESC RPE untreated
H1-ESC RPE + 5 µm 2AI
1µg/mL plamitoleic std
8.74 min
0
8.5
9
9.5
10
10.5
11
11.5
12
12.5
13
13.5
14
14.5
Acquisition Time (min)
H1-ESC RPE 5 µM 2AI
255.2333
253.2178
269.2491
283.2647
248.9728
281.2487
235.9543
241.2172
251.2022
267.2334
274.0601
279.2332
291.1660
H1-ESC RPE untreated
269.2485
255.2325
283.2638
253.2168
281.2481
248.9720
235.9524
241.2169
251.2005
267.2318
274.0593
279.2320
291.1602
Counts vs. Mass-to-Charge (m/z)
